# What motivates farmers to adopt low-carbon agricultural technologies? Empirical evidence from thousands of rice farmers in Hubei province, central China

**DOI:** 10.3389/fpsyg.2022.983597

**Published:** 2022-11-18

**Authors:** Linli Jiang, Haoqin Huang, Surong He, Haiyang Huang, Yun Luo

**Affiliations:** ^1^School of Economics and Management, Wuyi University, Jiangmen, China; ^2^National Post-Doctoral Innovation (Jiangmen) Demonstration Center, Jiangmen, China

**Keywords:** low-carbon agriculture, low-carbon technology, structural equation modeling, psychological mechanism, rice farmer

## Abstract

Low-carbon agriculture is essential for protecting the global climate and sustainable agricultural economics. Since China is a predominantly agricultural country, the adoption of low-carbon agricultural technologies by local farmers is crucial. The past literature on low-carbon technologies has highlighted the influence of demographic, economic, and environmental factors, while the psychological factors have been underexplored. A questionnaire-based approach was used to assess the psychological process underlying the adoption of low-carbon agricultural technologies by 1,114 Chinese rice farmers in this paper, and structural equation modeling (SEM) was empirically employed to test our theoretical model. The results indicated that farmers’ low-carbon production attitude and behavioral efficiency perception directly and positively affected the adoption of low-carbon agricultural technologies and indirectly affected it via low-carbon production intention. Besides, production implementation cost and socio-environmental factor could moderate the direct effects of low-carbon production attitude, behavioral efficiency perception, and low-carbon production intention on farmers’ adoption of low-carbon agricultural technologies. In this respect, socio-environmental factor yielded more significant moderating effects. Additionally, this research provides policy implications for promoting low-carbon agricultural technologies in developing countries and regions.

## Introduction

A low-carbon economy represents a novel approach implemented by many countries for economic development and protection against global warming (Jiang et al., 2010; He, 2016). As a developing country, China pledged to reduce carbon dioxide emissions per unit of GDP by 60–65% by 2030 compared with 2005 and included this as a restrictive indicator in the medium and long-term planning of national economic and social development at the Paris Climate Change Conference (Yang and Teng, 2018). Notwithstanding that excessive industrial greenhouse gas emissions are one of the leading causes of global warming, agricultural greenhouse gas emissions also threaten the global climate. According to the “Special Report on Climate Change and Land” published by the Intergovernmental Panel on Climate Change, agriculture, forestry, and other land use account for nearly 23% of the greenhouse gas emissions of human activities globally (IPCC, 2019), and the problems of chemical fertilizers and pesticide residues, soil compaction, food, and environmental safety caused by high-carbon agriculture have exacerbated (Maraseni et al., 2018). In fact, promoting low-carbon agricultural technologies can effectively mitigate global warming. Importantly, the decarbonization of farmers’ practices is key to promoting the low-carbon development of agriculture (Scarlat et al., 2015). Thus, investigating the psychological mechanism underlying farmers’ adoption of low-carbon agricultural technologies is of great theoretical and practical significance for developing countries to protect against global warming and foster sustainable agricultural economics.

Extant research on low-carbon production generally includes three aspects. The first one is the evaluation of low-carbon agricultural production. For example, Bai et al. (2019) calculated the production efficiency of low-carbon agriculture from the perspective of carbon emissions and sequestration to explore the impact of climate change on agricultural production. Moreover, Liu et al. (2020) constructed an evaluation index system for low-carbon agricultural production based on supply capacity, resource utilization, environmental quality, ecosystem maintenance, and farmers’ lives. The second aspect involves the determining factors associated with farmers’ adoption of low-carbon technologies. By applying regression models, such as Logistic and Probit, scholars comprehensively assessed the effects of the demographic, family characteristics, environment, and risk factors on farmers’ adoption of low-carbon technologies (Jain and Rekha, 2017; Liu et al., 2019; Zhang et al., 2019), and explored the consistency of low-carbon production intention and behavior regarding straw returning (Li et al., 2021b). Furthermore, studies have shown that low-carbon perception, value perception, and social norms significantly influence farmers’ adoption of low-carbon agricultural technologies (Jiang et al., 2018; Yu et al., 2018). The last aspect is intervention policies for farmers’ adoption of low-carbon technologies. A study pointed out that subsidies or a reasonable carbon tax contributed to reducing agricultural carbon emissions and promoting the development of low-carbon agriculture (Fan and Dong, 2018). Therefore, governments have the onus to actively promote low-carbon agriculture. Various measures (such as formulating subsidy policies for low-carbon agricultural production, constructing agricultural irrigation infrastructure, and promoting land-use rights transfer) can be undertaken by governments to foster low-carbon production among farmers (Pradhan et al., 2017; Zhu et al., 2018).

In some developing countries, low-carbon agricultural materials (such as soil testing formula fertilizer and biological pesticide) and technologies (such as intermittent irrigation and straw returning) with well-documented emission reduction effects have been gradually promoted in major agricultural production areas (Liu et al., 2019; Li et al., 2021b). However, the adoption rates of low-carbon agricultural materials and technologies remain low among farmers. Although farmers are willing, the adoption behavior is rarely observed, which is described as the phenomenon of “high intention—low behavior” (Vande Velde et al., 2018). Little emphasis has hitherto been placed on such psychological and behavioral phenomena, with most studies focused on the impact of demographic, economic, and environmental factors on farmers’ adoption of low-carbon technologies (Jain and Rekha, 2017; Liu et al., 2019; Zhang et al., 2019). Interestingly, social media has an essential impact on farmers’ adoption of low-carbon agricultural technologies (Yang et al., 2021), and farmers of the same clan usually participate in the same agricultural activities (Jiang et al., 2022). In addition, a psychological study showed that individual behavior is affected by environmental factors, and psychological factors such as cognition, emotion, and intention play an essential role (Strack et al., 2016). Accordingly, this paper intends to examine the psychological and situational factors that influence farmers’ decision-making on adopting low-carbon technologies. Rice is widely acknowledged as a food crop that significantly emits greenhouse gases during its growth period (Maraseni et al., 2018), thus rice farmers were selected as the study subject in this study. In order to reduce greenhouse gas emissions from the production process of rice farmers, this study aims to address the following questions: (1) What are the psychological factors that determine the adoption of low-carbon technologies by rice farmers? (2) What is the influence mechanism of these factors on their adoption behavior? (3) How to effectively guide rice farmers to participate in low-carbon rice production? These answers provide the basis for developing countries to effectively promote low-carbon agricultural technologies to protect the global climate.

## Theoretical background and hypotheses

### Adoption of low-carbon technologies

The adoption of low-carbon technologies by rice farmers refers to the use of low-carbon agricultural materials and management measures to reduce agricultural greenhouse gas emissions and improve the agricultural ecological environment (Liu et al., 2020). In this paper, based on the actual situation of low-carbon rice production in Hubei province of China, low-carbon technologies with the best carbon emission reduction effects, namely two low-carbon agricultural materials (i.e., soil testing formula fertilizer and biological pesticide) and two field management measures (i.e., intermittent irrigation and straw returning), were selected based on the opinions of 20 agronomy and crop science experts.

### Low-carbon production attitude

According to the theory of planned behavior (TPB), an attitude is a positive or negative evaluation of a given behavior (Ajzen and Fishbein, 1977), and a positive attitude can increase the probability of the behavior occurring (Li et al., 2021a). In this study, the low-carbon production attitude refers to rice farmers’ cognition and evaluation of climate change, low-carbon agriculture, and environmental protection, including low-carbon cognition and environmental awareness. An increasing body of evidence suggests that the more positive attitude of farmers towards low-carbon production technologies, the more likely their behavioral intentions are to improve (van Dijk et al., 2016; Mingolla et al., 2019; Waseem et al., 2020). Besides, some studies highlighted that the positive attitude of farmers positively impacts their behavioral efficiency perception and production behavior. For example, Bagheri et al. (2019) indicated that farmers’ attitude toward pesticides significantly affected their perception and behavior. Environmental awareness also positively affects their pro-environment production behavior (Zeng et al., 2019). Herein, rice farmers’ behavioral efficiency perception was divided into two aspects: the value perception of economic and ecological benefits (i.e., value perception) and the self-efficacy of adopting low-carbon agricultural technologies (i.e., self-efficacy).

Accordingly, rice farmers’ cognition and evaluation of climate change and low-carbon agriculture and their environmental awareness of soil, water quality, atmosphere, and other surrounding environments impact their intention and adoption of low-carbon technologies. Furthermore, there may be a close relationship between rice farmers’ attitude toward adopting low-carbon technologies and the perceived efficiency of adoption behavior. Consequently, rice farmers’ low-carbon production attitude (i.e., low-carbon cognition, environmental awareness) significantly affects their behavioral efficiency perception (i.e., value perception, self-efficacy), low-carbon production intention, and adoption of low-carbon technologies. The following hypotheses were proposed:

H1a: Low-carbon cognition of rice farmers has a positive effect on their value perception.

H1b: Low-carbon cognition of rice farmers has a positive effect on their self-efficacy.

H1c: Low-carbon cognition of rice farmers has a positive effect on their low-carbon production intention.

H1d: Low-carbon cognition of rice farmers has a positive effect on their adoption of low-carbon technologies.

H2a: Environmental awareness of rice farmers has a positive effect on their value perception.

H2b: Environmental awareness of rice farmers has a positive effect on their self-efficacy.

H2c: Environmental awareness of rice farmers has a positive effect on their low-carbon production intention.

H2d: Environmental awareness of rice farmers has a positive effect on their adoption of low-carbon technologies.

### Behavioral efficiency perception

According to the rational behavior theory and TPB, individual behavior efficiency perception or perceived behavior control can predict behavioral intention and implementation (Schifter and Ajzen, 1985; Ajzen and Timko, 1986; McCaul et al., 1988; Adnan et al., 2019). Moreover, a stronger perceptual capability of individual behavior efficiency has been associated with a greater likelihood of behavioral intention (de Lauwere et al., 2012). Interestingly, it has been reported that the intention of rice farmers to adopt low-carbon agricultural technologies was positively affected by their perception of the rice planting experience (Li et al., 2021a). Besides, perceived efficacy fostered farmers to produce in a pro-environment manners (Zeng et al., 2019). In this research, behavioral efficiency perception refers to rice farmers perceiving the effects of low-carbon agricultural technologies and their ability to implement them, including the value perception and self-efficacy. For example, during low-carbon rice cultivation, farmers may perceive the ecological value of low-carbon agricultural technologies to improve the environment and the convenience of using low-carbon agricultural materials. Traditional agricultural production technologies pollute the ecological environment and seriously threaten farmers’ health due to pesticide residues in agricultural products. The pressure on farmers to protect their health drives their learning of low-carbon agricultural production technologies and the value of these technologies in improving the ecological environment and solving food safety problems (Vuong, 2021, 2022). Current evidence suggests that farmers are more likely to apply organic fertilizers if they perceive the ecological and economic value in reducing environmental pollution (Li and Wu, 2021). Meanwhile, the loss aversion of farmers can reportedly harm their behavioral perceptions due to the increased economic costs of sustainable management measures (Mingolla et al., 2019). Therefore, there may be a close relationship between farmers’ perceived value and self-efficacy in the low-carbon production scenario.

On these grounds, rice farmers’ value perception of low-carbon agricultural technologies (e.g., environmental value, economic value, emission reduction value), as well as self-efficacy (information acquisition, purchase of agricultural materials, labor-saving) may positively affect their low-carbon production intention and adoption of low-carbon technologies (Borges and Lansink, 2016). Consequently, rice farmers’ behavioral efficiency perception (i.e., value perception, self-efficacy) significantly affects their low-carbon production intention and adoption of low-carbon technologies. The following hypotheses were proposed:

H3a: Value perception of rice farmers has a positive effect on their self-efficacy.

H3b: Value perception of rice farmers has a positive effect on their low-carbon production intention.

H3c: Value perception of rice farmers has a positive effect on their adoption of low-carbon technologies.

H4a: Self-efficacy of rice farmers has a positive effect on their low-carbon production intention.

H4b: Self-efficacy of rice farmers has a positive effect on their adoption of low-carbon technologies.

### Low-carbon production intention

Based on the TPB, individual behavioral intention positively affects the occurrence of behavior (Ajzen, 1991). A positive correlation has been documented between farmers’ production intention and actual production behavior (Lalani et al., 2016). However, other studies suggested a paradox between farmers’ behavioral intention and actual behavior; although farmers may have a firm behavior intention, it is not necessarily translated into the actual production behavior (Sharifzadeh et al., 2017; Hyland et al., 2018; Vande Velde et al., 2018). This discrepancy between intention and behavior may be caused by internal and external factors, such as production implementation cost and socio-environmental factor. Therefore, there may be a positive or negative causal effect between rice farmers’ low-carbon production intention and adoption of low-carbon technologies (Ajzen, 1991; Borges and Lansink, 2016). The following hypothesis was proposed:

H5: Low-carbon production intention of rice farmers may significantly affect their adoption of low-carbon technologies.

### Production implementation cost and socio-environmental factor

According to the above analysis, farmers’ attitude, behavioral efficiency perception, and intention will influence their adoption of low-carbon technologies. However, some studies have emphasized that farmers’ behavioral intention may not translate into actual behavior under the influence of some moderating variables (Hagger et al., 2002; Han, 2015). This phenomenon reveals that the decision of farmers to adopt low-carbon production is influenced by a series of factors, such as economic factors (Lo, 2014), policy conditions (Malawska and Topping, 2016), and family background (Aydogdu and Yenigün, 2016; Zhou et al., 2020). This paper mainly investigated the impact of production implementation cost and socio-environmental factor on rice farmers’ adoption of low-carbon agricultural technologies.

First, production implementation cost indicates the cost of adopting low-carbon agricultural technologies, including traditional farming habit and risk tolerance. Low-carbon agricultural materials and production technologies may increase investment and risk, leading to enhanced production costs and discouraging farmers from adopting low-carbon production (Liu et al., 2021). Meanwhile, the environmental value of adopting these low-carbon agricultural technologies cannot be directly exchanged for monetary value because of the lack of a new eco-surplus culture where the value created for the environment is rewarded with money (Vuong, 2021), which may reduce farmers’ enthusiasm for adoption. An increasing body of evidence suggests that economic compensation improves farmers’ environmental attitude (Burton et al., 2007; Koundouri et al., 2009). Besides, economic compensation can directly improve farmers’ attitude and indirectly affect their behavior by moderating the intensity of their behavioral intention (Castillo et al., 2021). Meanwhile, Liu et al. (2021) found that government subsidies positively moderated the effect of farmers’ risk perception on adopting groove ridge planting and subsoiling. Therefore, the production implementation cost may affect farmers’ adoption of low-carbon technologies by moderating their low-carbon production attitude, behavioral efficiency perception, and low-carbon production intention.

Second, it is widely acknowledged that farmers live in social groups, and their behavior is affected by the territorial socio-environmental factors. In this study, emphasis was placed on the cultural background and social environment of rice farmers, including the cultural background of small-scale farmers, government-led promotion, and group effect. Some researchers found that external environmental factors can change farmers’ behavior (Hagger et al., 2002). For example, significant differences were found between farmers’ perception level and decision-making behavior in different social organizations (such as cooperatives) (Han, 2015). At the same time, farmers’ social norm positively moderated the effect of individual norm on their organic fertilizer application behavior (Lo, 2014). Moreover, the perceived social pressure of individual farmers was closely related to their attitude and intention (Aydogdu and Yenigün, 2016; Malawska and Topping, 2016). Undeniably, if farmers obtain relevant information but remain skeptical about its significance, they may still not use or adopt low-carbon agricultural technologies. Therefore, the trust associated with the group effect plays an important role in the mindsponge-based information process of farmers (Vuong and Napier, 2015; Vuong et al., 2022). The above analysis confirms that local socio-environmental factors affect farmers’ attitude, perception, and intention toward low-carbon technologies for agriculture. Accordingly, the following hypotheses were proposed:

H6a: Production implementation cost moderates the effect of rice farmers’ low-carbon production attitude on their adoption of low-carbon technologies.

H6b: Production implementation cost moderates the effect of rice farmers’ behavioral efficiency perception on their adoption of low-carbon technologies.

H6c: Production implementation cost moderates the effect of rice farmers’ low-carbon production intention on their adoption of low-carbon technologies.

H7a: Socio-environmental factor moderates the effect of rice farmers’ low-carbon production attitude on their adoption of low-carbon technologies.

H7b: Socio-environmental factor moderates the effect of rice farmers’ behavioral efficiency perception on their adoption of low-carbon technologies.

H7c: Socio-environmental factor moderates the effect of rice farmers’ low-carbon production intention on their adoption of low-carbon technologies.

### Theoretical framework

Overall, from the perspective of the psychological mechanism of information processing, rice farmers’ adoption of low-carbon technologies results from a mindset change where the perceived value of such behavior is integrated into their mindset, which can be seen as a mindsponge-based information process (Vuong and Napier, 2015). Indeed, rice farmers adopt low-carbon technologies when subjectively perceived as beneficial. Otherwise, the idea will be rejected. A positive net value is obtained after farmers consider all related costs and benefits that they are aware of, which results in a change of their intention into actual behavior when the net perceived value of the act reaches a certain threshold (individual-specific). Accordingly, rice farmers’ mindset about the perceived value of adopting low-carbon agricultural technologies can be changed by improving their low-carbon cognition and environmental awareness. Meanwhile, through the mindsponge-based information process, relevant information about the value perception and self-efficacy of low-carbon agricultural technologies is integrated into rice farmers’ mindset, leading to changes in their adoption intention and behavior. Moreover, the production implementation cost and socio-environmental factor play key roles in rice farmers’ adoption intention turning into behavior by influencing the perceived costs and benefits.

**FIGURE 1:**
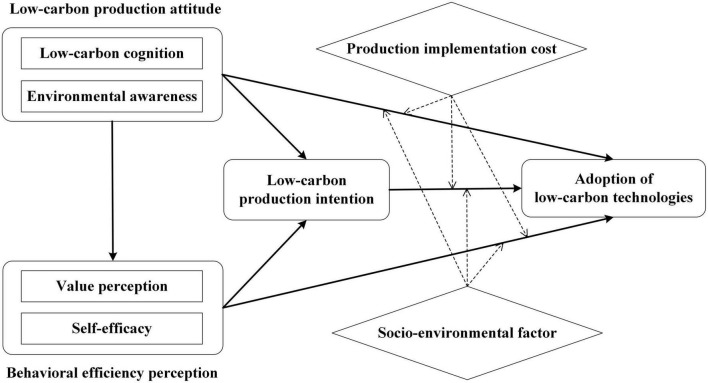
Theoretical framework of rice farmers’ adoption of low-carbon technologies.

Consequently, the theoretical framework of this research was established (Figure 1).

## Materials and methods

### Data collection

**FIGURE 2:**
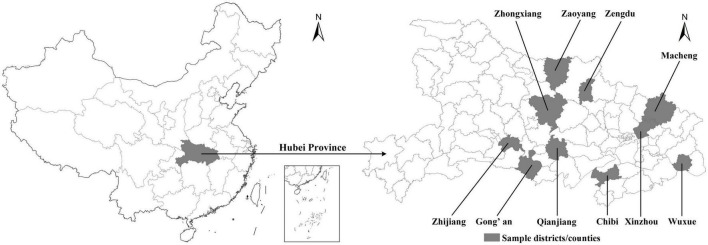
Survey region and sample distribution.

Hubei province, well-established as the top rice-producing region with the highest output in China, was used as the representative area for investigating Chinese rice farmers’ adoption of low-carbon technologies. We conducted a questionnaire survey based on the theoretical model (see Figure 1) in three major rice-growing areas in Hubei province, encompassing 10 districts/counties: Zaoyang, Zhongxiang, and Zengdu in the north; Zhijiang, Gong’an, Qianjiang, and Chibi in the south; and Macheng, Xinzhou, and Wuxue in the east (see Figure 2). After these 10 cultivating districts/counties were chosen, the survey team randomly selected two to three towns in each district/county and two to three villages in each town. Then 20 rice farmers were randomly selected from the lists of villagers provided by the village officials. After excluding invalid questionnaires, 1,114 questionnaires were eligible for analysis, with the validity rate of 92.83%.

### Questionnaire design

As mentioned earlier, this research investigated rice farmers’ low-carbon production attitude in terms of low-carbon cognition and environmental awareness. Regarding rice farmers’ low-carbon cognition, this study was based on the scales of Maloney et al. (1975), Guagnano et al. (1995), and Oliver and Rosen (2010). The measurement items were modified according to the research purpose. To fit the environmental characteristics of China, this paper measured rice farmers’ environmental awareness from three dimensions: soil pollution, water pollution, and air pollution. The scales were based on the research results of Maloney et al. (1975), Dunlap et al. (2000), and De Groot and Steg (2009). Meanwhile, farmers’ behavioral efficiency perception was studied in terms of value perception and self-efficacy. First, farmers’ value perception was measured from three dimensions, namely environmental value, economic value, and emission reduction value. The measurement items of these three variables were based on the scale of Steg et al. (2014). Moreover, rice farmers’ self-efficacy was based on the scale of Fielding et al. (2008), measured from three dimensions: access to information, purchase of agricultural materials, and labor-saving. The original measurement items were adjusted appropriately based on the current situation in rural China and the characteristics of rice farmers. Furthermore, in line with the Chinese rural culture, this paper designed the scales for rice farmers’ low-carbon production intention and adoption of low-carbon technologies based on findings reported by Stern et al. (1999) and Folse et al. (2010).

To conform to the characteristics of Chinese rural culture, this paper assessed the production implementation cost from two aspects: traditional farming habit and risk tolerance. The traditional farming habit investigated the costs of transformation, including deep plowing habit and extensive production mode. Moreover, the risk tolerance included rice farmers’ judgment on climate change risk and their risk preference for adopting low-carbon technologies. Furthermore, many small-scale rice farmers in China were managed by village committees. Therefore, the social environment factor was measured from three aspects: cultural background of small farmer, government-led promotion, and influence of group effect. Among them, the cultural background of smallholders included self-discipline consciousness and conservative mentality. Besides, the government-led promotion contained subsidy promotion and punishment regulation. Finally, the influence of group effect was associated with conformity psychology and convergence behavior.

After completing the draft of the questionnaire, the researchers revised it twice. First, the experts related to agronomy evaluated the rationality of the questionnaire, and the researchers adjusted the content according to the evaluation results. Next, a preliminary survey was conducted on representative rice farmers and village cadres, and the researchers modified the questionnaire measurement items according to the survey results. The final measurement items related to rice farmers’ adoption of low-carbon technologies are shown in Table 1.

**TABLE 1:** Scales of rice farmers’ adoption of low-carbon technologies and relevant variables.

Variable/dimension		Num.	Item	Range
Low-carbon cognition	Climate change	A1	True or False: (1) Excessive greenhouse gas emissions lead to a global average temperature drop. (2) Greenhouse gas is carbon dioxide. (3) Burning straw will not cause climate change. (4) Using chemical fertilizers will produce greenhouse gas emissions. (5) Climate change will threaten agricultural production.	0 = incorrect answers; 1 = correct answer; sum: 0 ∼ 5.
	Low-carbon technologies	A2	I know low-carbon agricultural technologies (such as intermittent irrigation and straw returning).	5-point Likert scale: 1 = strongly disagree;
	Low-carbon agriculture	A3	Low-carbon agriculture is a low-pollution, low-emission production method.	2 = disagree; 3 = neutrality; 4 = agree; 5 = strongly agree.
Environmental awareness	Soil pollution	B1	I will pay attention to the soil salinization and lack of organic matter on arable land.	
	Water pollution	B2	I will pay attention to the pollution of ponds and groundwater in the village.	
	Air pollution	B3	I will pay attention to air pollution and air quality.	
Value perception	Environmental value	C1	Using fewer chemical fertilizers and pesticides is good for the environment and soil.	
		C2	I must reduce environmental pollution from agricultural production.	
	Economic value	C3	Taking the lead in adopting low-carbon agricultural technologies will increase my income in the future.	
	Emission reduction value	C4	I think low-carbon agricultural technologies can effectively reduce agricultural greenhouse gas emissions.	
Self-efficacy	Access to information	D1	I can easily get information about low-carbon agricultural technologies.	
	Purchase of agricultural materials	D2	I can easily buy low-carbon materials such as soil testing formula fertilizers and biological pesticides.	
	Labor-saving	D3	Using low-carbon fertilizers and pesticides is very convenient and labor-saving for me.	
Low-carbon production intention	Promotion	Y1	I am willing to adopt the low-carbon rice production technologies and management measures if they are promoted.	
	New technologies	Y2	I am willing to try new agricultural technologies or management approaches.	
	Demonstration	Y3	I would like to adopt low-carbon rice production technologies if they are demonstrated.	
	Subsidy	Y4	I intend to adopt low-carbon agricultural technologies if the government grants ecological subsidies.	
Adoption of low-carbon technologies	Agricultural material application	Z1	I use soil testing formula fertilizer in the process of rice planting.	5-point Likert scale:
		Z2	I use biological pesticides in the process of rice planting.	1 = never; 2 = seldom; 3 = sometimes; 4 = usually; 5 = always.
	Field management	Z3	I irrigate intermittently in the process of rice planting.	
	Waste disposal	Z4	I return the straw to the field when the rice is harvested.	
Traditional farming habit	Deep plowing habit	E1	I always profoundly plow rice fields.	5-point Likert scale:
	Extensive production mode	E2	There is no need to change the extensive production mode that relies on overusing fertilizers and pesticides.	1 = strongly disagree; 2 = disagree; 3 = neutrality; 4 = agree; 5 = strongly agree.
Risk tolerance	Risk level	F1	I think extreme weather phenomenon has a significant impact on rice yield.	
	Risk preference	F2	If all farmers adopt low-carbon agricultural technologies, I think they will be more reliable.	
Small farmer cultural background	Self-discipline	G1	Even if the government provides a suggested farming method, I will cultivate the land as I wish.	
	Conservation	G2	I wouldn’t believe in new agricultural technologies easily.	
Government-led promotion	Subsidy	H1	I won’t adopt low-carbon agricultural technologies without government subsidies.	
	Punishment	H2	Under the strict penalties of the government, I will return the straw to the field.	
Group effect	Conformity	I1	I will follow most people in adopting low-carbon agricultural technologies.	
	Convergence	I2	My behavior is more easily influenced by those around me.	

There are five questions that examine the objective perception of rice farmers on climate change (A1).

### Methodology

Structural equation modeling (SEM) was constructed to empirically test the theoretical framework of farmers’ adoption of low-carbon technologies. Besides, the mediating effect of low-carbon production intention was verified by causal steps approach, and the moderating effects of production implementation cost and socio-environmental factor were tested by hierarchical regression. The specific methods were described as follows:

#### Structural equation modeling

**FIGURE 3:**
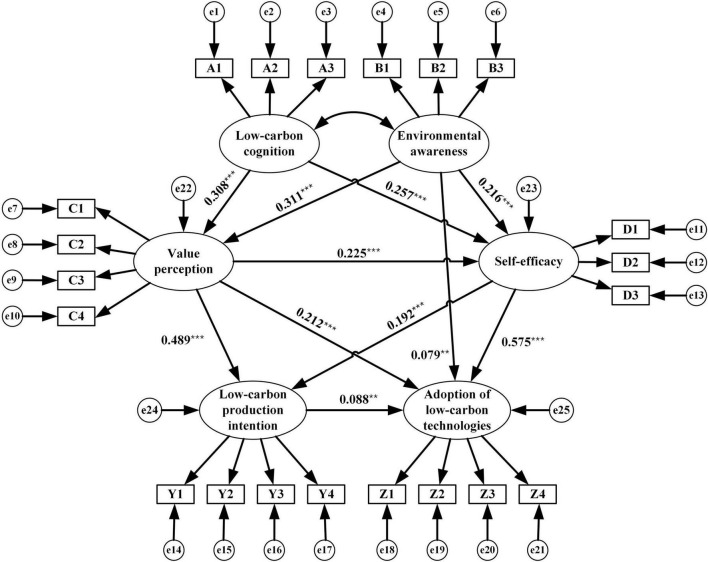
Path model and standardized factor loadings. ** and *** represent significant at 5% and 1%, respectively.

This study employed SEM to explore the latent relationships between rice farmers’ adoption behavior and influencing factors (see Figure 3). SEM is composed of the measurement model and structural model. Equations (1) and (2) are measurement models used to test the relationships between latent variables (exogenous and endogenous latent variables) and observational variables. Equation (3) is a structural model that can test the causal effects between exogenous and endogenous latent variables.


(1) $$\text{X}={\text{Λ}}_{X}\,\xi +\text{δ}$$

(2) $$\text{Y}={\text{Λ}}_{y}\,\text{η}+\varepsilon$$

(3) η=B⁢η+Γ⁢ξ+ζ

where X represents the exogenous latent variable vector, including rice farmers’ low-carbon cognition, environmental awareness, value perception, and self-efficacy; ξ represents the observational variables of the exogenous latent variables; Y represents the vector of the endogenous latent variable, reflecting the low-carbon production intention and the adoption of low-carbon technologies; η represents the observational variables of the endogenous latent variables. Λ_*X*_ and Λ_*y*_ represent the correlation coefficient matrix between exogenous latent variables, endogenous latent variables, and their corresponding observational variables, respectively; δ and ε are the measurement error vectors of the exogenous and endogenous observational variables; B represents the matrix of structural coefficients among endogenous latent variables, reflecting the mutual influence between endogenous latent variables (i.e., the effect of rice farmers’ low-carbon production intention on the adoption of low-carbon technologies); Γ is the structure coefficient matrix between exogenous and endogenous latent variables, indicating the causal effect of exogenous latent variables X (low-carbon cognition, environmental awareness, value perception, and self-efficacy) on endogenous latent variables Y (low-carbon production intention and adoption of low-carbon technologies); and ζ is the random error vector of the structural equation.

#### Causal steps approach

Causal steps approach has been widely used to test the effectiveness of mediating variables in the social science field. In this study, causal steps approach was utilized to examine the mediating effect of low-carbon production intention. There are three steps to judge the mediating effect: the first is to test the relationship between key independent variables (i.e., low-carbon cognition, environmental awareness, value perception, and self-efficacy) and dependent variable (i.e., adoption of low-carbon technologies); the second is to examine the effect of key independent variables on the mediating variables; and the third is to analyze the effects of key independent variables and mediating variables on the dependent variable. In addition, we can calculate the marginal effect of the key independent variables to compare the strength of the role of mediating variable.

#### Hierarchical regression

Hierarchical regression is a multiple regression method used to determine the order of different variables in the regression equation theoretically or according to the actual needs of researchers. In this study, hierarchical regression was utilized to examine the moderating effect of situational variables (i.e., production implementation cost and socio-environmental factor). Specifically, explanatory variables, moderating variables, and interaction terms were successively introduced into the regression model. A comparison of the three models’ square sum of partial regression was conducted to determine whether moderating variables and interaction terms significantly affect the dependent variables. Moreover, a moderating effect was observed when the moderating variable and interaction term significantly affected the dependent variables.

## Results

### Descriptive analysis of sample characteristics

The rice farmers’ socio-demographic and farm characteristics are shown in Table 2. The respondents were predominantly male (70.11%) and aged from 51 to 60 (39.77%). Most respondents (37.43%) had junior high school education background (with 7∼9 years of education). Besides farming, 29.44% of farmers held part-time jobs. Agriculture represented a long-term occupation for 31–40 years for 34.20% of farmers. 20.38% of farmers indicated that most household earnings came from agricultural production. Overall, the sample distribution of the socio-demographic and farm characteristics were in line with the actual situation, which means the sample was representative of the information of rice farmers in Hubei province.

**TABLE 2:** Basic characteristics of the surveyed rice farmers.

Variable	Item	Frequency	Percentage	Variable	Item	Frequency	Percentage
Gender	Male	781	70.11	Part time job	Yes	328	29.44
	Female	333	29.89		No	786	70.56
Age	≤40	57	5.12	Educational level (years)	0∼3	192	17.24
	41∼50	287	25.76		4∼6	334	29.98
	51∼60	443	39.77		7∼9	417	37.43
	61∼70	278	24.96		10∼12	153	13.73
	≥71	49	4.40		≥13	18	1.62
Farming experience (years)	≤20	138	12.39	Agricultural income proportion	≤20%	316	28.36
	21∼30	263	23.61		21∼40%	253	22.71
	31∼40	381	34.20		41∼60%	175	15.71
	41∼50	254	22.80		61∼80%	143	12.84
	≥51	78	7.00		≥81%	227	20.38

### Low-carbon production status

According to the survey results (see Table 3), straw returning was the most adopted technology, with 84.74% of the rice farmers (*n* = 944) implementing this technology. Moreover, the average cognition level of straw returning was significantly higher than other low-carbon agricultural technologies, substantiating that straw burning prohibition and comprehensive utilization in Hubei province achieved remarkable achievements. Besides, 33.66% of rice farmers (*n* = 375) sprayed abamectin-containing biological pesticide, and 30.79% (*n* = 343) adopted intermittent irrigation. However, only 17.68% (*n* = 197) of farmers adopted the soil testing formula fertilizer.

**TABLE 3:** Rice farmers’ cognition level and adoption of low-carbon agricultural technologies.

Low-carbon agricultural technology	Cognition level from 1 to 5		Status of adoption	
	Sample	Average	Frequency	Percentage
Soil testing formula fertilizer	1,114	2.45	197	17.68%
Biological pesticide	1,114	2.77	375	33.66%
Intermittent irrigation	1,114	2.67	343	30.79%
Straw returning	1,114	3.19	944	84.74%

### Reliability and validity testing

SPSS 22.0 and AMOS 22.0 were used to test the reliability and validity of the questionnaire measurement scale (see Table 4). The overall Cronbach’s α coefficients of the latent variables (i.e., environmental awareness, value perception, self-efficacy, low-carbon production intention, and adoption of low-carbon technologies) were superior to 0.6, suggesting good consistency and reliability of the scale. Although the overall Cronbach’s α coefficient of low-carbon cognition was 0.592, it is acceptable for social sciences research (Peterson, 1994). According to the validity analysis, the Kaiser-Meyer-Olkin (KMO) value of all latent variables exceeded 0.6, and Bartlett’s sphericity tests were significant at the 1% statistical level, indicating that the scale has good construct validity. Besides, the measured items were suitable for confirmatory factor analysis (CFA).

**TABLE 4:** Results of the variable reliability and validity analysis.

Variable	No.	Standardized factor loading	Cronbach’s α	KMO value	Bartlett’s sphericity test		Composite reliability (CR)
					χ^2^	*p*	
Low-carbon cognition (LC)	A1	0.413	0.592	0.612	340.287	0.000	0.600
	A2	0.758					
	A3	0.532					
Environmental awareness (EA)	B1	0.531	0.741	0.626	855.889	0.000	0.759
	B2	0.880					
	B3	0.714					
Value perception (VP)	C1	0.586	0.765	0.777	1063.914	0.000	0.775
	C2	0.658					
	C3	0.778					
	C4	0.694					
Self-efficacy (SE)	D1	0.713	0.766	0.674	901.415	0.000	0.718
	D2	0.659					
	D3	0.661					
Low-carbon production intention (LPI)	Y1	0.852	0.709	0.751	770.877	0.000	0.744
	Y2	0.476					
	Y3	0.459					
	Y4	0.767					
Adoption of low-carbon technologies (ALT)	Z1	0.443	0.652	0.707	588.303	0.000	0.660
	Z2	0.525					
	Z3	0.614					
	Z4	0.695					

CFA was used to test the convergent validity of the scale (see Table 4). The results showed that the standardized factor loadings of most observational and latent variables exceeded 0.5. Although some observational variables’ standardized factor loadings were slightly smaller (between 0.4 and 0.5), they were deemed acceptable (Ford et al., 1986). Additionally, all standardized factor loadings were significant at the 1% statistical level, showing that the scale has good convergent validity. Meanwhile, each latent variable’s composite reliability (CR) was calculated to judge the internal quality and a CR value of a latent variable greater than 0.6 suggested good reliability of the measurement model and high consistency of the factor constructs. As shown in Table 4, the CR values of latent variables were not less than 0.6, demonstrating that the model has good internal quality. Besides, correlations were compared with the square root of AVE values. Table 5 presents the descriptive statistics of the key variables, including the square root of AVE values, means, standard deviations, and correlation coefficients. Since all correlations were smaller than the respective square root of AVE values, the discriminant validity was supported (Fornell and Cha, 1994; Hair et al., 2014). Overall, these results substantiate the reliability and validity of the measurement model.

**TABLE 5:** Descriptive statistics of key variables.

Variable	LC	EA	VP	SE	LPI	ALT
LC	**0.586**					
EA	0.324***	**0.722**				
VP	0.383***	0.369***	**0.683**			
SE	0.274***	0.313***	0.314***	**0.678**		
LPI	0.216***	0.246***	0.474***	0.297***	**0.662**	
ALT	0.238***	0.313***	0.378***	0.518***	0.294***	**0.577**
Mean	2.405	3.390	3.659	3.132	3.797	2.392
Standard deviation	0.795	1.106	0.842	1.098	0.693	0.898

This table presents the descriptive statistics of the key variables, including the square root of AVE values, means, standard deviations, and correlation coefficients (*** represents significant at 1%). Square roots of AVE values are shown in bold on matrix diagonal.

### Model fitting

To solve the issue of Chi-square value (χ^2^) expansion driven by large samples, the ratio between Chi-square to the degree of freedom (χ^2^/*df*) was selected as an indicator of fitness (Hair et al., 2010). The results of the goodness of fit test were as follows: N (sample) = 1,114, χ^2^/df = 2.351 (<3), GFI = 0.969 (>0.90), AGFI = 0.953 (>0.90), CFI = 0.969 (>0.90), NFI = 0.947 (>0.90), IFI = 0.969 (>0.90), TLI = 0.957 (>0.90), RMSEA = 0.035 (<0.08), CN (0.01) = 609 (>200). The results suggested a good consistency between the theoretical model and the practical data. After deleting insignificant paths between latent variables according to the modification indices, the standardized path coefficient are shown in Figure 3 and Table 6.

**TABLE 6:** Path coefficients of the structural equation modeling.

Path	Standard path coefficient	SE	CR	*P*
Low-carbon cognition → Value perception	0.308	0.110	5.890	***
Low-carbon cognition → Self-efficacy	0.257	0.118	5.034	***
Low-carbon cognition → Low-carbon production intention	–	–	–	–
Low-carbon cognition → Adoption of low-carbon technologies	–	–	–	–
Environmental awareness → Value perception	0.311	0.048	7.595	***
Environmental awareness → Self-efficacy	0.216	0.058	4.780	***
Environmental awareness → Low-carbon production intention	–	–	–	–
Environmental awareness → Adoption of low-carbon technologies	0.079	0.049	1.983	**
Value perception → Self-efficacy	0.225	0.051	4.814	***
Value perception → Low-carbon production intention	0.489	0.046	11.706	***
Value perception → Adoption of low-carbon technologies	0.212	0.051	4.344	***
Self-efficacy → Low-carbon production intention	0.192	0.038	5.155	***
Self-efficacy → Adoption of low-carbon technologies	0.575	0.054	10.179	***
Low-carbon production intention → Adoption of low-carbon technologies	0.088	0.038	2.176	**

** and *** represent significant at 5% and 1%, respectively.

The estimation results showed that rice farmers’ low-carbon cognition significantly affected their value perception and self-efficacy, with standardized path coefficients of 0.308 and 0.257, respectively. Rice farmers’ environmental awareness was positively correlated with their value perception, self-efficacy, and adoption of low-carbon technologies, with standardized path coefficients of 0.311, 0.216, and 0.079, respectively. Moreover, the standardized path coefficients of rice farmers’ value perception on their self-efficacy, low-carbon production intention, and the adoption of low-carbon technologies were 0.225, 0.489, and 0.212, respectively. Furthermore, rice farmers’ self-efficacy was positively correlated with low-carbon production intention and adopting low-carbon technologies, with path coefficients of 0.192 and 0.575, respectively. Finally, the standardized path coefficient of rice farmers’ low-carbon production intention on their adoption of low-carbon technologies was 0.088. Overall, the main effects of the variables in the theoretical model were validated. Additionally, the mediating and moderating effects were further verified by causal steps approach and hierarchical regression.

### Mediating effect

To assess the mediating effect of low-carbon production intention (see Table 7), SPSS 22.0 was employed to centralize the observational variables corresponding to the six latent variables (i.e., low-carbon cognition, environmental awareness, value perception, self-efficacy, low-carbon production intention, and adoption of low-carbon technologies). Then, the mediating effect was analyzed with the mean values of each latent variable by causal steps approach. The results showed that rice farmers’ low-carbon cognition affected their adoption of low-carbon technologies through low-carbon production intention; that is, low-carbon cognition indirectly affected the adoption of low-carbon technologies. The ratio of the mediating effect to the total effect was 23.05%, which explained 24.70% of the variance in the dependent variable. Furthermore, other latent variables, namely environmental awareness, value perception, and self-efficacy, affected the adoption of low-carbon technologies through the partial mediating effects of low-carbon production intention; and the mediating effects of low-carbon production intention accounted for 18.16%, 18.56%, and 8.77%, and explained 22.36%, 12.65%, and 14.14% of the variance in the dependent variable, respectively.

**TABLE 7:** Mediating effect of the low-carbon production intention.

IV	M	DV	IV→DV	IV→M	IV+M→DV		Mediation type
					IV	M	
LC	LPI	ALT	0.238***	0.216***	0.183***	0.254***	Partial mediation
EA	LPI	ALT	0.313***	0.246***	0.256***	0.231***	Partial mediation
VP	LPI	ALT	0.378***	0.474***	0.308***	0.148***	Partial mediation
SE	LPI	ALT	0.518***	0.297***	0.472***	0.153***	Partial mediation

IV, M, and DV are independent variables, mediating variables, and dependent variables, respectively. *** represents significant at 1%.

### Moderating effect

According to the research design, there were two types of moderating variables, namely production implementation cost (i.e., traditional farming habit and risk tolerance) and the socio-environmental factor (i.e., small farmer cultural background, government promotion, and group effect). As previously described by Aiken et al. (1991), Cohen et al. (2003), and Hayes (2013), to make the coefficients of the regression equation more explanatory, the first step in the moderating effect test is to centralize the observational variables corresponding to the seven latent variables (i.e., low-carbon cognition, environmental awareness, value perception, self-efficacy, low-carbon production intention, production implementation cost, and socio-environmental factor). Then, the moderating effect was analyzed with the mean values of the centralized observational variables included in the above seven latent variables. Given that there is no need to centralize the dependent variable during moderation analysis, the mean value of the observational variables corresponding to rice farmers’ adoption of low-carbon technologies was introduced directly into the hierarchical regression model. Finally, independent variables, moderating variables, and interaction terms were introduced successively for hierarchical regression (see Table 8).

**TABLE 8:** Moderating effects of production implementation cost and socio-environmental factor.

Variable	[M_1_] = Production implementation cost			[M_2_] = Socio-environmental factor		
	Model 1	Model 2	Model 3	Model 1	Model 2	Model 3
(constant)	2.392***	2.393***	2.395***	2.392***	2.392***	2.374***
Low-carbon cognition	0.011	–0.022	–0.025	0.011	–0.019	–0.022
Environmental awareness	0.101***	0.088***	0.084***	0.101***	0.100***	0.089***
Value perception	0.180***	0.161***	0.161***	0.180***	0.180***	0.189***
Self-efficacy	0.409***	0.428***	0.434***	0.409***	0.425***	0.433***
Low-carbon production intention	0.060**	0.026	0.035	0.060**	0.034	0.035
Moderating variables [M*_*i*_*]		−0.141***	−0.133***		−0.098***	−0.103***
Low-carbon cognition × [M*_*i*_*]			0.002			–0.005
Environmental awareness × [M*_*i*_*]			0.021			0.068**
Value perception × [M*_*i*_*]			0.047			–0.044
Self-efficacy × [M*_*i*_*]			−0.060**			0.058**
Low-carbon production intention × [M*_*i*_*]			–0.040			−0.073**
Adjusted *R*^2^	0.328	0.344	0.345	0.328	0.336	0.343
Variation of *R*^2^	0.331	0.016	0.004	0.331	0.008	0.010

** and *** represent significant at 5% and 1%, respectively.

**FIGURE 4:**
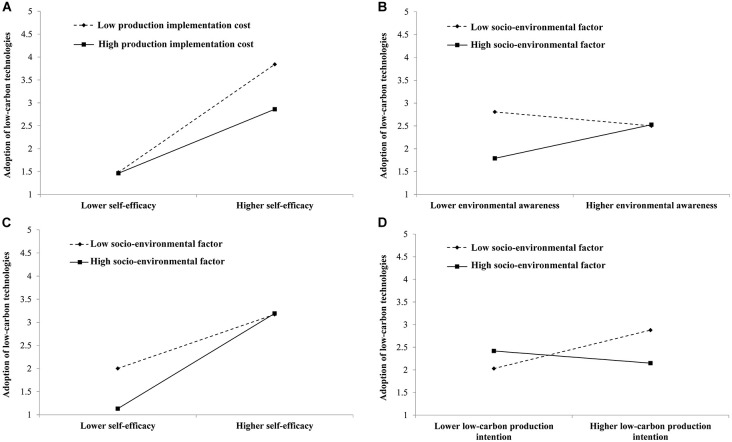
Moderating effects of production implementation cost and socio-environmental factor. **(A)** Production implementation cost moderates the effect of self-efficacy on the adoption of low-carbon technologies. **(B)** Socio-environmental factor moderates the effect of environmental awareness on the adoption of low-carbon technologies. **(C)** Socio-environmental factor moderates the effect of self-efficacy on the adoption of low-carbon technologies. **(D)** Socio-environmental factor moderates the effect of low-carbon production intention on the adoption of low-carbon technologies.

As shown in Table 8, the production implementation cost negatively moderated the effect of self-efficacy on farmers’ adoption of low-carbon technologies. Besides, socio-environmental factor positively moderated the effects of environmental awareness and self-efficacy on farmers’ adoption of low-carbon technologies but negatively moderated the effect of low-carbon production intention. Figure 4 depicts the moderating effects of production implementation cost and socio-environmental factor on the path of rice farmers’ adoption of low-carbon technologies. The essence of moderating effect is considering how it affects the relationship between independent and dependent variables when the moderating variable is at a high and a low level, respectively. As shown in Figure 4, if the effect value (i.e., the slope) of the high group (i.e., when the moderating variable is at the high level) was greater than the effect value of the low group (i.e., when the moderating variable is at the low level), it exerted an enhancement effect on the pathway of “independent variable → dependent variable,” and vice versa.

As seen in Figure 4A, a robust positive moderating effect was observed between self-efficacy and adoption of low-carbon technologies for rice farmers with low production implementation cost, suggesting that improving their self-efficacy perception contributed to promoting the adoption of low-carbon technologies. However, for rice farmers with high production implementation cost, self-efficacy slightly affected their adoption of low-carbon technologies. It could be seen that the production implementation cost had an interferential effect on the pathway of “self-efficacy → adoption of low-carbon technologies.” Thus, reducing the cost or risk of adopting low-carbon agricultural technologies would contribute to the positive transformation from farmers’ self-efficacy perception to actual adoption behavior.

We found that environmental awareness strongly affected the adoption of low-carbon technologies for rice farmers with high socio-environmental factor, suggesting that improving their environmental awareness can effectively encourage them to engage in low-carbon production (see Figure 4B). Besides, the effect was dramatically diminished for rice farmers with low socio-environmental factor. It could be seen that the socio-environmental factor had an enhancement effect on the pathway of “environmental awareness → adoption of low-carbon technologies,” suggesting that green and low-carbon social environment could induce better environmental awareness of rice farmers, and they were more likely to be engaged in low-carbon production. Accordingly, the socio-environmental factor played a vital catalytic role in farmers’ adoption of low-carbon agricultural technologies.

Compared to rice farmers with low socio-environmental factor, the positive effect of self-efficacy on adopting low-carbon technologies was more potent for rice farmers with high socio-environmental factor (see Figure 4C). Accordingly, the socio-environmental factor reinforced the effect of self-efficacy on farmers’ adoption of low-carbon technologies. It could be seen that socio-environmental factors enhanced the pathway of “self-efficacy → adoption of low-carbon technologies.” In other words, a positive social environment for low-carbon agriculture could strengthen rice farmers’ self-efficacy, making them more likely to adopt low-carbon technologies. Accordingly, if a social environment advocating low-carbon agricultural production was established via the local government, it would contribute to the positive transformation from farmers’ self-efficacy to actual adoption behavior.

For rice farmers with low socio-environmental factor, low-carbon production intention positively influenced their adoption of low-carbon technologies (see Figure 4D). Consequently, enhancing those farmers’ low-carbon production intention could effectively promote their low-carbon production participation, compared with rice farmers with high socio-environmental factor. Importantly, it can be seen that the socio-environmental factor exerted an interferential effect on the pathway of “low-carbon production intention → adoption of low-carbon technologies,” accounting for the “high intention—low behavior” phenomenon. In fact, for rice farmers lacking low-carbon agricultural material knowledge and professional equipment for field management, their adoption of low-carbon technologies remained challenging even when they had subjective intents (Wang et al., 2018). This finding applied to farmers’ first exposure to these new agricultural materials and production equipment, such as biological pesticide, soil testing formula fertilizer, intermittent irrigation facility, etc.

## Discussion

### Theoretical contributions

In the present study, the adoption rates of soil testing formula fertilizer, biological pesticide, intermittent irrigation, and straw returning by sample rice farmers were 17.68%, 33.66%, 30.79%, and 84.74%, respectively. This finding suggested that the straw burning ban and comprehensive utilization management in Hubei province achieved remarkable achievements, but the adoption rates of other low-carbon agricultural technologies were dismal. Over the years, much emphasis has been placed on the effects of demographic (Jain and Rekha, 2017), economic (Liu et al., 2019), and environmental factors (Zhang et al., 2019) on farmers’ adoption of low-carbon technologies. Overwhelming evidence substantiates that psychological factors can change farmers’ adopting behavior (Jiang et al., 2018; Yu et al., 2018), and a comprehensive analysis framework based on farmers’ production practices has not been established to date. To fill this gap, our study comprehensively assessed farmers’ adoption of low-carbon technologies from four aspects, namely soil testing formula fertilizer, biological pesticide, intermittent irrigation, and straw returning, and constructed a theoretical model of rice farmers’ adoption of low-carbon technologies. Furthermore, based on questionnaires from Chinese rice farmers (*n* = 1,114), the theoretical model was empirically tested by SEM.

Importantly, we found that when farmers lacked basic cognition of climate change, low-carbon technologies, and environmental awareness, they could not perceive the value of adopting low-carbon technologies, further hindering intention formation and the actual adoption behavior. Compared to the literature (Jiang et al., 2018; Yu et al., 2018), our theoretical model based on rice farmers’ low-carbon production practice enriched the multidimensional connotation of farmers’ low-carbon production attitude and behavioral efficacy, and comprehensively explained the psychological factors driving the adoption of low-carbon technologies.

Meanwhile, growing evidence suggests that unique abilities and circumstances often limit the transformation from individual intention to actual behavior, described as the phenomenon of “high intention—low behavior” (Vande Velde et al., 2018). Unlike these previous studies (Sharifzadeh et al., 2017; Hyland et al., 2018), this paper confirms and extends this finding from the practical level. Rice farmers’ environmental awareness, self-efficacy, and low-carbon production intention will be moderated and restricted by situational factors (i.e., production implementation cost and socio-environmental factor). The high costs or risks of adopting low-carbon agricultural technologies can inevitably impede the transformation of rice farmers’ self-efficacy perception into actual adoption behavior. Creating a green and low-carbon social environment can enhance the effects of environmental awareness and self-efficacy on rice farmers’ adoption of low-carbon technologies. Subsequently, rice farmers were more likely to engage in low-carbon production. However, it should be borne in mind that socio-environmental factor has a significant negative moderating effect on the pathway of “low-carbon production intention → adoption of low-carbon technologies.” It indicates that the effect of low-carbon production intention on the adoption of low-carbon technologies is less substantial due to farmers’ lack of knowledge on low-carbon production (about biological pesticide and soil testing formula fertilizer) or the external resources conditions (of technical guidance, intermittent irrigation facilities, etc.) for field management although the social environment may help improve the subjective intents of farmers.

### Policy implications

This research provides four critical connotations for the policymakers of developing countries that aim to popularize low-carbon agriculture to protect the global climate. First of all, since the low-carbon production attitude of farmers has a positive effect on the adoption of low-carbon technologies mediated by the effect of low-carbon production intention, improving farmers’ attitude and intention can effectively promote low-carbon agricultural technologies. Low-carbon agricultural knowledge and technical training are key to improving farmers’ attitude toward low-carbon production and increasing low-carbon production intention. Governments of developing countries can improve the popularization of low-carbon agricultural knowledge with the help of new media platforms. Importantly, intensive, comprehensive, and sustainable low-carbon agricultural knowledge propaganda is a reasonable way to activate and arouse farmers’ intention toward low-carbon production from the psychological level.

Secondly, besides the direct positive effect, farmers’ behavioral efficiency perception also positively affects the adoption of low-carbon technologies mediated by low-carbon production intention. Therefore, improving farmers’ behavioral efficiency perception is necessary for promoting low-carbon agriculture. Specifically, agricultural research institutes can refine low-carbon technologies such as intermittent irrigation and straw returning and provide low-carbon agricultural production guidance to enhance farmers’ confidence and perception of efficiency in implementing low-carbon production.

Moreover, given that the implementation costs of low-carbon agriculture hinder farmers from adopting low-carbon agricultural technologies, government subsidies may be a solution. Indeed, in most developing countries, agricultural income is the primary source for farmers, and agricultural production costs are critical to their livelihoods. Accordingly, improving the subsidies for low-carbon agriculture and reducing the low-carbon production costs of farmers are paramount approaches to promoting low-carbon agriculture. Furthermore, ecological compensation should be incorporated into the agricultural subsidies, which is beneficial to reduce the direct and hidden costs of low-carbon production for farmers and stimulate their enthusiasm for participating in low-carbon production.

Last but not least, the production activities of farmers occur in a specific social environment. Therefore, the need for establishing a green and low-carbon social environment should be emphasized. A green and low-carbon social environment will induce environmental awareness and higher self-efficacy in farmers, who are more likely to engage in low-carbon production. However, socio-environmental factor negatively moderates the effect of farmers’ low-carbon production intention on the adoption of low-carbon technologies, which leads to the phenomenon of “high intention—low behavior” of farmers in low-carbon production. More precisely, although the low-carbon production social environment may help improve the subjective intention of farmers, the effect of low-carbon production intention on the adoption of low-carbon technologies will be less significant if farmers live in an environment bereft of knowledge of low-carbon agricultural materials and professional field management equipment. Consequently, low-carbon agricultural information promotion and low-carbon agricultural technologies training are crucial for individual farmers and critical to creating a green and low-carbon social environment, which fosters farmers to carry out low-carbon production. At the same time, the infrastructure, machinery, and equipment required for low-carbon agriculture are heavy financial burdens for farmers. The low-carbon agricultural production loan policies and agricultural materials subsidies are practical methods to reduce these costs.

### Limitations and further research

The empirical results reported herein should be considered in light of some limitations. As shown above, rice cultivation is the main occupation of most farmers, and rice yield and economic returns are of great importance to them. Whether farmers finally adopt low-carbon agricultural technologies depends on psychological factors (i.e., low-carbon production attitude, behavioral efficiency perception, and low-carbon production intention) and the actual financial benefits. In addition, the financial benefits of adopting those technologies can affect farmers’ psychological perceptions of low-carbon agriculture. Given that the returns on investment of farmers’ adoption behavior were not studied in this paper, we could not analyze how the economic benefits of adopting low-carbon agricultural technologies affect the psychological changes of farmers. Based on the above analysis, we believe that the psychology related to farmers’ adoption behavior of low-carbon agricultural technologies is a topic worth exploring in the future. Moreover, experimental data could be used to validate the theoretical model proposed in this paper. Finally, the cost and benefit data of low-carbon production can be accurately collected in field experiments to further study the mechanism underlying the influence of economic factors affecting psychological cognition and the actual behavior of farmers.

## Conclusion

The previous literature on farmers’ adoption of low-carbon technologies has highlighted demographic, economic, and environmental factors (Jain and Rekha, 2017; Liu et al., 2019; Zhang et al., 2019), while farmers’ psychology has been largely underexplored. Our results substantiated that farmers’ low-carbon production attitude and behavioral efficiency perception directly and positively affected the adoption of low-carbon agricultural technologies and indirectly affected it via low-carbon production intention. Therefore, more supportive policies are warranted to improve farmers’ low-carbon production attitude and behavioral efficiency perception. Moreover, the direct effects of low-carbon production attitude, behavioral efficiency perception, and low-carbon production intention on farmers’ adoption of low-carbon agricultural technologies were moderated by production implementation cost and socio-environmental factor. In this respect, socio-environmental factor yielded more significant moderating effect. This observation corroborates that advocating a social environment for low-carbon agricultural production is essential for improving farmers’ adoption behavior which could be harnessed to develop new policies to foster farmers’ adoption of low-carbon technologies.

## Data availability statement

The original contributions presented in the study are included in the article/supplementary material, further inquiries can be directed to the corresponding author/s.

## Ethics statement

The studies involving human participants were reviewed and approved by the National Natural Science Foundation of China (Project No. 72003051) and the Ministry of Education of Humanities and Social Science of China (Project No. 19YJC790048). Written informed consent for participation was not required for this study in accordance with the national legislation and the institutional requirements.

## Author contributions

LJ conceived and designed the study and performed the empirical analysis of survey data. HQH contributed significantly to manuscript preparation. SH, HYH, and YL was put forward valuable idea and participated in wrote the manuscript. All authors contributed to the article and approved the submitted version.
